# Antimicrobial effects of copper nanoparticles with green tea and neem formulation

**DOI:** 10.6026/97320630018284

**Published:** 2022-03-31

**Authors:** Anjali Anna Thomas, Remmiya Mary Varghese, S Rajeshkumar

**Affiliations:** 1 Department of Orthodontics and Dentofacial Orthopaedics, Saveetha Dental College and Hospital, Saveetha Institute Of Medical and Technical Sciences, Saveetha University, 162, Poonamallee high road, Chennai - 600077, Tamil Nadu, India; 2 Nanobiomedicine Lab, Department of Pharmacology, Saveetha Dental college and Hospital, Saveetha Institute Of Medical And Technical Sciences, Chennai - 600077, Tamil Nadu, India

**Keywords:** Copper, nanoparticles, neem, green tea, plant extract, antimicrobial activity

## Abstract

Nanotechnology is the science which is about manipulating matter, atom by atom and is associated with particles smaller than 100 nm in size. Copper nanoparticles are used mainly due to its surplus amount, low cost, easy availability and biocompatible property. Green synthesis of copper nanoparticles is very simple, economical and eco-friendly method that does not involve any toxic chemicals. The aim of our study is green synthesis of copper nanoparticles using green tea and neem formulation and assessment of its antimicrobial effects. 20mM of copper sulphate solution is mixed with 40mL of plant extract and 60 mL of distilled water was added and made it into 100 ml solution. Once the copper nanoparticles are synthesized the solution is characterized using UV- vis-spectroscopy and was scanned in double beam UV-vis- spectrophotometer from 300 nm to 700nm wavelength. The antimicrobial property of copper nanoparticle is evaluated by agar well diffusion method. The colour change from green to brown and peak observed in UV-vis- spectrophotometer was associated with the synthesis of copper nanoparticles. Copper nanoparticle from green tea and tea extract has good antimicrobial activity against *S.mutans*, *C.albicans*, *E.faecalis*, & *S.aureus*. Copper nanoparticles can be efficiently synthesised from green and neem formulation. These copper nanoparticles showed good antibacterial properties and are effective against oral pathogens.

## Background:

Nanotechnology is the science which is about manipulating matter, atom by atom [1]. Nanotechnology is associated with particles smaller than 100 nm in size [2 - check with author]. Due to its small size and high surface area, nanoparticles increase the state of activity. The size of nanoparticles depends on the method of reduction and its surrounding environment. Nanodentistryis the science and technology of maintaining good oral health through the use of nonmaterial's including tissue engineering and nanorobotics [3]. Currently nanoparticles are of in use in various fields of biomedical and pharmaceutical like diagnostics, biomarkers, bio-imaging, cosmetics, antibacterial, anticancer, immunology, cardiology, genetic engineering, drug delivery for treating cancer and other infectious diseases [4].Due to their small size, physical and chemical properties, high surface area for interaction and wide area of application of metal nanoparticles are increasing [5 - check with author]. Copper nanoparticles are used in the field of nanotechnology and Nanomedicine due to their good optical, electrical and anti-fungal/bacterial properties [6 - check with author]. Copper nonmaterial's are been used mainly due its abundant amount, low cost and availability when compared to other metals such as silver and gold. Copper is the basic and biocompatible element that has good therapeutic and antibacterial property known since a very long time [7]. The copper nanoparticle was synthesized by various methods such as metal vapours synthesis [8] laser irradiation [9], exploding wire method [10], vacuum vapour deposition, and microemulsion [11]. But these techniques are of high cost and involve the use of toxic chemicals. The herbal preparation plays an immense role in synthesizing the nanoparticles naturally. Nanoparticles synthesized from plant extract are able to overcome many toxic effects of conventional methods [12]. Green synthesis of nanoparticle is a very simple, economical, eco-friendly and repeatable method which does not require intense energy, pressure, temperature, or toxic chemicals [13]. The previous studies showed that the antimicrobial and anticancer activities of nanoparticles synthesized from plant extract are better [14]. The green synthesis technique usually uses water, biological extracts, biological systems, and microwave-assisted synthesis which are non toxic and eco-friendly. There are many methods of green synthesis of nanoparticles. The commonly used methods for green synthesis are using biological routes using micro organisms such as bacteria, yeast, plant and animal extracts and enzymes or their by products [15 - check with author]. These are most commonly used because they are eco -friendly, non-toxic, cost efficient, and mild conditions. The objective of study was green synthesis of copper nanoparticles using green tea (Camellia Sinensis) and neem formulation and assessment of its antimicrobial effects.

## Materials and methods:

### Preparation of Plant Extract:

Green tea and neem were purchased from the herbal care center.2 grams of green tea and 2 grams of neem were measured and added to a conical flask and were dissolved in 100ml ml of distilled water. Then the extract was heated at 60°C for 7-8 minutes in a heating mantle. Using a blotting paper the extract was then filtered into another conical flask using a Whatmann No.1 filter paper. The filtrate which is in the conical flask is needed plant extract.

### Synthesis of Silver Nanoparticles:

20 mill moles of copper sulphate is mixed with 40 mL of plant extract and 60 mL of distilled water in a conical flask. The flask was then placed in the orbital shaker at 65 rpm and then in a magnetic stirrer at 450 rpm for uniform distribution. The color change was noted regularly with an interval of one hour for two days. With the help of UV spectroscopy the prepared copper containing green tea and neem extract was recorded to check for the synthesis of nanoparticle. It was then subjected to centrifugation at 10,000 rpm for 10 mins in a centrifuge. The copper nanoparticle pellets were then collected to perform antimicrobial activity tests.

### Characterization of nanoparticles:

Once the nanoparticles are synthesized the solution is characterized by using UV- vis-spectroscopy.3ml of the solution is taken in cuvette and scanned in double beam a UV- visible spectrophotometer (ELICO SL 210 UVV is spectrophotometer) from 300 nm to 700 nm wavelengths and the results were recorded graphically.

### Antimicrobial activity of copper nanoparticle:

The antimicrobial efficiency of copper nanoparticles was assessed using agar well diffusion method. The antibacterial activity of copper nanoparticles tested against four different bacterial isolates like *E. Facalis*, Streptococcus mutans, Streptococcus aureus and Candida albicans. Fresh bacterial cultures were prepared on the surface of Muller-Hinton agar plates in a broth medium. Different concentrations of copper nanoparticles (25, 50, and 100 µL) were incorporated into the wells and the plates were incubated at 37°C for 24 hours. The antibiotic discs (ampicillin) were used as control. After incubation, the zone of incubation formed around the discs was measured and notes down.

## Results:

### Visual observation:

Nanoparticles have a great interest due their unique optical properties. During their process of synthesis they exhibit a different range of colors. The plant extract contains various phytochemicals that converts the copper sulphate into copper nanoparticles identified by the colour change. This colour change from green to brown is due to the formation of copper nanoparticles (Figure 1 - see PDF).

### UV-visible Spectroscopy:

The copper nanoparticles were synthesized using copper sulphate and green and neem extract displays an absorption peak at 340 nm (Figure 2 - see PDF).This peak was associated with the synthesis of copper nanoparticles. The broadened SPR peak that was observed in the UV-visible spectrum confirmed that polydisperse nanosized particles are synthesized [16].

### Antibacterial activity of copper nanoparticles against oral pathogens:

Copper has an excellent antimicrobial activity against a wide range of oral pathogens and this property is enhanced with its nanoparticle form [17]. The zone of growth inhibition of cells is because of the distraction of cell membrane by copper nanoparticles, which leads to the breakdown of cell enzymes [14]. The results of our study reveals that the green tea and neem mediated copper nanoparticles showed effective antibacterial activity (Figure 3 & 4 - see PDF).

## Discussion:

Copper as a metal is well known for its antimicrobial and anti-inflammatory properties since several years [18 - check with author]. The copper nanoparticles can be easily synthesized and is cost effective material so it can be an alternative for gold and silver nanoparticles [19]. Currently, environmental friendly synthesis of copper nanoparticles has gained much interest [20]. Green synthesis of nanoparticles is more trending as the plant itself acts as both reducing and capping agent also because of its eco-friendly [21]. Green tea (Camellia sinensis) contains many phytoconstituents like epigallocatechin-3-gallate (EGCG), theanins, catechins and polyphenols and is reported to have neuroprotective activity [22]. It is reported that they even prevent proliferation of carcinoma cells [23]. Green tea is considered as a reducing agent for the synthesis of the different morphology of copper nanoparticles because it contains a high amount of polyphenols and other organic groups in it. The reduction mechanism happens in two steps initially when the precursor is added a complex is formed by breaking the -OH bond and a partial bond with a metal ion is formed. Then there is transfer of electrons wherein the metal ions gets reduced to nanoparticles by the breakage of the partial bond, and then it get oxidized to ortho-quinone [24]. Green synthesis of copper nanoparticles using green tea and neem formulation is a simple, economical, eco-friendly process. In our study the colour change indicates the synthesis of copper nanoparticles which is in accordance with the previous studies [25]. In general UV-vis spectra can be used for assessing the size and shape-controlled nanoparticles in the aqueous solution with 200-800nm wavelength range [26]. In our study it was observed that the copper nanoparticles have good antimicrobial properties against oral pathogens. The zone of inhibition is increased with the increase in concentration of copper nanoparticles. These results were homogeneous when CuNPs synthesized using glycerol-polyvinyl alcohol [27], polyurethane with silver and copper nanoparticles [28] copper based additives [29] and copper-resistant Bacillus cereus [30].

## Conclusion:

Copper nanoparticles were efficiently synthesized from green and neem formulation. The use of toxic chemicals is avoided since the nanoparticles are synthesized using green synthesis method, which is non toxic, economical and eco friendly. These copper nanoparticles showed good antibacterial properties also. Since it is effective against the oral pathogens and they can be used in toothpaste and oral medicines. Hence the copper nanoparticles could be expected to be used in future for the effective drug systems and immunity against diseases.

## Funding:

This research did not receive any specific grant from funding agencies in the public, commercial, or not-for-profit sectors.

## Ethical clearance:

Ethical clearance taken from Saveetha University, ethical approval number is IHEC/SDC/ORTHO-1902/21/286.

## References

[1] Whitesides GM, Christopher L (2001). J Scientific American.

[3] Freitas Jr RA (2000). Journal of the American Dental Association (1939).

[4] Ganapathy D (2020). Journal of Evolution of Medical and Dental Sciences.

[7] Panigrahi S (2004). Journal of Nanoparticle Research.

[8] Grace M (2009). Journal of Engineered Fibers and Fabrics.

[9] Yeh MS (1999). The Journal of Physical Chemistry.

[10] Li M (2013). Chinese Journal of Chemistry.

[11] Yallappa S (2013). Spectrochim Acta A Mol Biomol Spectrosc..

[12] Rajeshkumar S, Bharath LV (2017). Chemico-Biological Interactions,.

[13] Agarwal H (2018). Chemico-Biological Interactions.

[14] Sivaraj R (2014). Spectrochim Acta A Mol Biomol Spectrosc..

[16] Ramyadevi J (2011). Materials Letters.

[17] Grass G (2011). Appl. Environ. Microbiol.

[19] Rajeshkumar S, Rinitha G (2018). Open Nano.

[20] Rajeshkumar S (2019). Journal of Photochemistry and Photobiology B: Biology.

[21] Rajeshkumar S, Nail P (2018). Biotechnology Reports.

[22] Kakuda T (2002). Biological & Pharmaceutical Bulletin.

[23] Takada M (2002). Pancreas.

[24] Gottimukkala KSV (2017). Journal of Nanomedicine & Biotherapeutic Discovery.

[25] Gunalan S (2012). Molecular & Biomolecular Spectroscopy,.

[26] Ananth A (2015). Chemical Engineering Journal.

[27] Dobrovolny K (2017). Journal of Alloys and Compounds.

[28] https://www.springer.com/series/8898.

[29] Palza H (2018). International Journal of Antimicrobial Agents.

[30] Tiwari M (2016). Process Biochemistry.

